# Supplementary material for: Molecular Characterisation of Cryptosporidium spp. in Mozambican Children Younger than 5 Years Enrolled in a Matched Case-Control Study on the Aetiology of Diarrhoeal Disease
Source: Pathogens. 2021 Apr 9;10(4):452. doi: 10.3390/pathogens10040452 (PMC8070020; doi:10.3390/pathogens10040452)
Supplement: Supplementary file 1 [file pathogens-10-00452-s001.zip › Table S2_Messa_et_al_Pathogens_2021.docx]

**Table S2.** Diversity and frequency of *Cryptosporidium* family subtypes within *C. hominis* (subtype family I), *C. parvum* (subtype family II) and *C. meleagridis* (subtype family III) in asymptomatic (non-cases) children under 5 years of age according to severity of clinical manifestations, age group, and HIV coinfection. Children were recruited during the Global Enteric Multicenter Study at the Manhiça district (Maputo, southern Mozambique), 2007–2012. Figures between brackets represent relative frequencies.

|  |  | ***C. hominis*** | | | | ***C. parvum*** | | | | ***C. meleagridis*** |
| --- | --- | --- | --- | --- | --- | --- | --- | --- | --- | --- |
| **Variable** | **Total**  **(*n* = 60)** | **Ia**  **(*n* = 16)** | **Ib**  **(*n* = 13)** | **Ie**  **(*n* = 12)** | **If**  **(*n* = 4)** | **IIb**  **(*n* = 1)** | **IIc**  **(*n* = 8)** | **IIe**  **(*n* = 2)** | **IIg**  **(*n* = 1)** | **IIIb**  **(*n* = 3)** |
| **Matched group** |  |  |  |  |  |  |  |  |  |  |
| MSD | 47 (78.3) | 13 (82.3) | 8 (61.5) | 11 (91.7) | 4 (100) | 1 (100) | 6 (75.0) | 2 (100) | 0 (0.0) | 2 (66.7) |
| LSD | 13 (21.7) | 3 (18.7) | 5 (38.5) | 1 (8.3) | 0 (0.0) | 0 (0.0) | 2 (25.0) | 0 (0.0) | 1 (100) | 1 (33.3) |
| **Age (months)** |  |  |  |  |  |  |  |  |  |  |
| 0–11 | 25 (41.7) | 5 (31.2) | 7 (53.9) | 5 (41.7) | 2 (50.0) | 1 (100) | 3 (37.5) | 1 (50.0) | 0 (0.0) | 1 (33.3) |
| 12–23 | 32 (53.3) | 10 (62.5) | 4 (30.8) | 7 (58.3) | 2 (50.0) | 0 (0.0) | 5 (62.5) | 1 (50.0) | 1 (100) | 2 (66.7) |
| 24–59 | 3 (5.0) | 1 (6.3) | 2 (15.4) | 0 (0.0) | 0 (0.0) | 0 (0.0) | 0 (0.0) | 0 (0.0) | 0 (0.0) | 0 (0.0) |
| **Co-infections** |  |  |  |  |  |  |  |  |  |  |
| HIV+^1^ | 0 (0.0) | 0 (0.0) | 0 (0.0) | 0 (0.0) | NA | NA | 0 (0.0) | NA | NA | NA |

^1^ Frequencies calculated over the total of HIV+ children only.

HIV: Human immunodeficiency virus; LSD: Less severe diarrhoea; MSD: Moderate-to-severe diarrhoea. NA: Not applicable.
