# Supplementary figures and images for: Molecular Characterisation of Cryptosporidium spp. in Mozambican Children Younger than 5 Years Enrolled in a Matched Case-Control Study on the Aetiology of Diarrhoeal Disease
Source: Pathogens. 2021 Apr 9;10(4):452. doi: 10.3390/pathogens10040452 (PMC8070020; doi:10.3390/pathogens10040452)

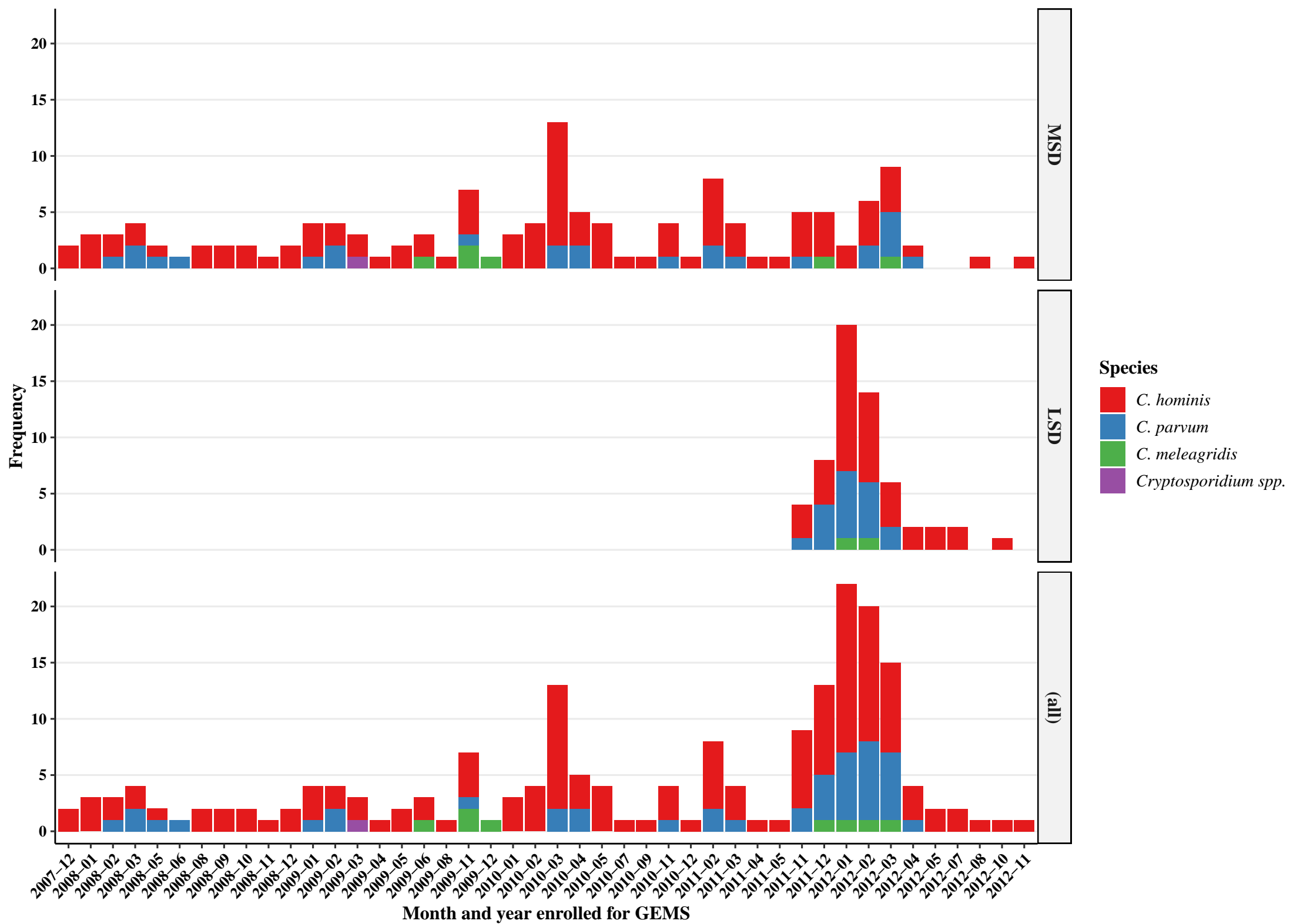

Supplement: Supplementary file 1 [file pathogens-10-00452-s001.zip › Figure_S1_Messa_et_al_Pathogens_2021.pdf]

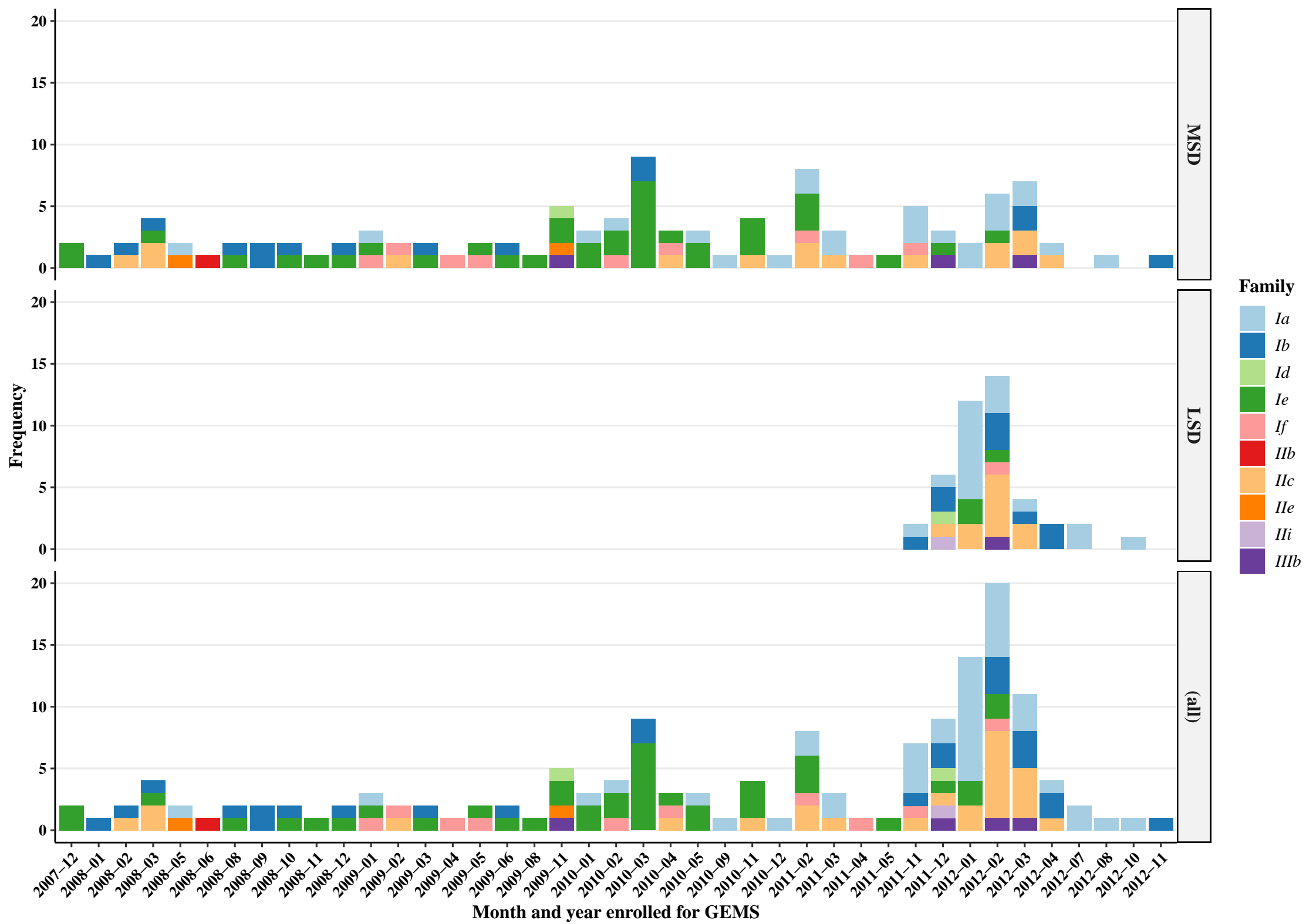

Supplement: Supplementary file 1 [file pathogens-10-00452-s001.zip › Figure_S2_Messa_et_al_Pathogens_2021.pdf]
